# Lactobacilli Probiotics Prevent Amyloid-Beta Fibril Formation In Vitro

**DOI:** 10.1007/s12602-025-10776-z

**Published:** 2025-10-13

**Authors:** Sanaa Harrass, Michael Quansah, Sachin Kumar, Michael Radzieta, Bhawantha Jayawardena, Christopher Jones, Monique David, Benjamin Heng, Liam D. H. Elbourne, Seth Amanquah, Patrick Adjei, Mario Capunzo, Silvana Mirella Aliberti, Slade O. Jensen, Mourad Tayebi

**Affiliations:** 1 School of Medicine, Western Sydney University, Campbelltown, NSW 2560 Australia; 2 University of Ghana Medical School/Korle Bu Teaching Hospital, Accra, Ghana; 3 South West Sydney Limb Preservation and Wound Research, Ingham Institute for Applied Medical Research, Liverpool, NSW Australia; 4 School of Science, Western Sydney University, Paramatta, NSW Australia; 5 Macquarie Medical School, Macquarie University, Sydney, NSW Australia; 6 ARC Centre of Excellence in Synthetic Biology, Macquarie University, Sydney, Australia; 7 Department of Medicine, Surgery and Dentistry “Scuola Medica Salernitana”, University of Salerno, Salerno, Italy

**Keywords:** Lactobacilli probiotics, Microbiome, Alzheimer’s disease, Amyloid β, Tau protein, Molecular docking, Protein aggregation

## Abstract

Alzheimer’s disease (AD) is characterized by the buildup of extracellular aggregated amyloid-β (Aβ) peptides, following sequential enzymatic cleavage of amyloid precursor protein, along with intraneuronal accumulation of hyperphosphorylated Tau proteins and subsequent neuronal loss. Despite extensive research, the precise mechanisms underlying Aβ and Tau-mediated neurodegeneration remain elusive. Inhibiting protein aggregation has been a primary focus for mitigating neuronal toxicity. Probiotics have emerged as a promising preventative measure against cognitive decline in AD, with several in vivo and clinical trials demonstrating the efficacy of select bacterial strains in slowing AD progression. However, these studies lack direct molecular evidence on the effects of probiotics on Aβ aggregation kinetic. Inhibiting protein aggregation is key to reducing neuronal toxicity. While probiotics have shown promise in preventing cognitive decline in Alzheimer’s disease, supported by in vivo and clinical studies, direct molecular evidence of their impact on Aβ aggregation kinetics remains lacking. In this study, we conducted bioinformatic and physicochemical assessments, including molecular docking of proteins derived from 13 probiotic strains against Aβ and Tau, identifying four strains predicted to efficiently inhibit Aβ aggregation. Kinetic studies confirmed that both the probiotic formulation and its derived supernatant significantly inhibited the conversion of monomeric Aβ and Tau into aggregated forms. To explore bioavailability, we administered the probiotic formulation to healthy individuals and detected its presence in stool samples, demonstrating survival through the gastrointestinal tract. These findings suggest that specific probiotic strains may serve as therapeutic candidates for targeting Aβ and/or Tau aggregation, with further studies warranted to assess their potential clinical utility in AD.

**Supplementary Information:**

The online version contains supplementary material available at 10.1007/s12602-025-10776-z.

## Introduction

This study presents a novel, multidisciplinary approach that integrates in silico screening, molecular docking, aggregation kinetics, and human probiotic survivability profiling to elucidate the mechanistic role of select probiotic strains in modulating Aβ and tau aggregation, addressing a critical gap in the current understanding of their therapeutic potential in Alzheimer’s disease.

Alzheimer’s disease (AD) is the most common cause of dementia, a neurodegenerative disorder characterized by memory loss and cognitive decline [1]. AD presents a significant global health challenge, affecting approximately 50 million individuals worldwide; a number that continues to rise due to the aging population and the lack of effective treatments [2–5].

The gut-brain axis, a bidirectional communication network between the gastrointestinal tract (GIT) and the brain, plays a crucial role in brain function and is increasingly recognized for its involvement in AD pathogenesis [6, 7]. Several mechanisms have been proposed to explain how alterations in gut microbiota may influence AD development, including neuroinflammation, Aβ and tau aggregation, neurotransmitter dysregulation, and oxidative stress [8–11].

Studies investigating the use of probiotics to modulate the gut microbiome in AD animal models and clinical trials have yielded promising results. Specifically, *Lactobacillus* and *Bifidobacterium* have shown notable effects [12, 13], with a combination of these strains demonstrating benefits through multiple mechanisms. For example, in AD animal models, *Lactobacillus* and *Bifidobacterium* were shown to produce neurotransmitters such as acetylcholine, regulate brain metabolites, modulate neuronal activity, and downregulate Aβ-induced gene expression [13–18]. Clinical trials further demonstrated that these probiotics could regulate serum metabolites and reduce oxidative stress in AD patients [17–19].

Probiotics confer additional benefits by producing short-chain fatty acids (SCFAs) and bacteriocins, and by stimulating the gut-brain axis and immune system [20]. SCFAs such as acetate, butyrate, and propionate, produced by specific gut bacteria, may influence brain function by modulating immune responses, endocrine signaling, and neuronal factors. Moreover, probiotics such as *Lactobacillus* and *Bifidobacterium* species have been shown to restore synaptic plasticity by normalizing long-term potentiation, enhancing signal transmission in the brain [21]. *Lactobacillus plantarum* has been found to reduce neurofibrillary tangles and amyloid plaques, while increasing acetylcholine levels in the hippocampus and cerebral cortex in AD animal models [13].

The role of probiotics in modulating the symptoms and progression of AD remains uncertain due to the limited number of clinical trials conducted to date. Among these, two studies have demonstrated that probiotic supplementation can improve cognitive function and metabolic status in AD patients [17–19]. While these findings suggest that probiotics may slow AD progression and positively impact cognition, larger-scale and longer studies are needed to establish a clearer relationship between microbial diversity, cognitive status, and disease progression in AD patients.

In this study, we aimed to evaluate the ability of specific *Lactobacillus* probiotic strains to inhibit Aβ and Tau aggregation and assess their presence in fecal samples following oral administration in healthy individuals. This component of the study, involving oral administration in healthy individuals, was conducted solely to evaluate the gastrointestinal survivability of the selected probiotic strains. It was not intended to assess clinical efficacy, but rather to confirm the strains’ ability to withstand the digestive environment and reach the intestine in viable form. Building on the current understanding of the gut-brain axis and the emerging role of probiotics in neurodegenerative disease, our investigation explores a novel avenue for targeting amyloidogenic processes at the interface between microbiota and brain health. The originality of our approach lies in the integration of in vitro and ex vivo analyses to determine both functional anti-amyloidogenic properties and the strains’ gastrointestinal survivability.

## Materials and Methods

### Probiotics Genome Assembly

#### Quality Control of Sequencing Data

Proprietary paired forward and reverse (R1 and R2) fastq files for 13 probiotic strains were obtained from Probiotics Australia (Ormeau, Australia). To evaluate the quality of the sequencing data, we utilized the FastQC v0.11.9 tool on the Galaxy platform (https://usegalaxy.org) to generate quality statistics, including per-base sequence quality, GC content, sequence duplication levels, and adapter contamination. This initial assessment allowed us to identify potential issues, such as poor-quality bases, overrepresented sequences, and adapter sequences that required removal. Subsequently, we employed the Trimmomatic v0.39 tool on Galaxy to trim low-quality reads and remove adapter sequences. Trimmomatic was run in paired-end mode using a sliding window approach, where a 4-base wide window scanned through each read and trimmed regions where the average quality dropped below a predefined threshold. Reads below a minimum length threshold were also discarded to ensure only high-quality sequences were retained [22]. FastQC was run again on the processed fastq files to compare the quality of reads before and after trimming. The post-trimming quality assessment confirmed improvements in sequence quality, as indicated by higher average Phred scores, reduced adapter contamination, and a more uniform GC content distribution. These steps ensured that only high-quality sequencing data were used for downstream analyses [23].

#### Genome Assembly Using Hybrid Approach

To assemble the probiotics’ DNA fragments obtained from high-throughput sequencing into complete genomes, the trimmed paired files were processed using the Unicycler pipeline v0.4.8 on the Galaxy platform. Unicycler is specifically designed for the hybrid assembly of small genomes (such as those in bacteria, viruses, or organelles), utilizing both short- and long-read sequencing data to produce complete and high-quality assemblies [24]. In this study, only short-read Illumina sequencing data were available; therefore, Unicycler was run in its short-read mode, which employs a SPAdes-based approach to iteratively assemble contigs and resolve repeat regions to the extent possible. De novo assembly was performed, meaning assembly was conducted without prior knowledge of a reference genome [25]. During assembly, Unicycler optimized the scaffolding of contigs and assessed assembly graphs to generate the most contiguous genome possible. The pipeline also performed automatic error correction to minimize misassemblies. The quality of the final assembly files generated by Unicycler was assessed using the Quast tool v5.0.2 on Galaxy. Quast provides various statistical measures for evaluating an assembly, including the total length of the assembly, the number of contigs or scaffolds, and the N50 and N75 measurements. A higher N50 value typically indicates a more contiguous assembly, reflecting longer, high-quality assembled sequences. Additionally, Quast generates three distinct plots: cumulative length of the contigs, Nx plot, and GC content plot [26]. The GC content plot was particularly useful in verifying the consistency of the assemblies with expected GC composition of the probiotic strains, while the Nx plot provided insights into the distribution of contig lengths.

#### Genome Annotation and Feature Identification

For protein identification within the genome sequences, we annotated the genomes using the Prokka pipeline v1.14.6 on Galaxy. Prokka is a computational tool designed for the rapid, automated annotation of features, including protein-coding regions and RNA genes in each sequence. The annotation process begins with genome sequence preprocessing, ensuring input files are properly formatted for Prokka. Annotation of protein-coding regions involves a two-step process: first, regions are identified using Prodigal, which scans the genome sequence to detect open reading frames (ORFs) based on coding potential and sequence composition; and second, the functions of the encoded proteins are predicted based on their similarity to proteins in various protein or protein domain databases. Prokka utilizes multiple curated databases, including UniProt, RefSeq, and TIGRFAMs, to assign functional annotations with high confidence. Additionally, it detects rRNA, tRNA, and other non-coding RNA genes using specialized tools such as RNAmmer and Aragorn. Prokka is capable of swiftly annotating genomes from probiotics, archaeal, and viral taxa, and it produces standardized output files in formats such as GenBank, EMBL, and GFF [27]. The generated annotations were systematically reviewed to confirm the presence of key functional genes relevant to probiotic activity, such as those involved in adhesion, stress resistance, and antimicrobial compound production. To identify and confirm the species and sub-strain level identity of the sequenced probiotic genomes, we employed Kraken2 v2.1.2, a high-throughput taxonomic classification tool optimized for metagenomic and genomic datasets. Kraken2 performs taxonomic assignments by mapping exact k-mer matches between query sequences and a comprehensive reference database, assigning reads based on the lowest common ancestor (LCA) algorithm.

For our analysis, the paired-end trimmed FASTQ files were used as input. Kraken2 was run on the Galaxy platform using the standard Kraken2 database (March 2023 build), which contains curated complete genomes from RefSeq for bacteria, archaea, and viruses. The classification process involved the following:

- *Database indexing*: Kraken2 breaks down each sequence into overlapping k-mers (default k = 35) and searches for exact matches in the database, recording all taxonomic identifiers associated with each k-mer [28].

- *Taxonomic assignment*: For each read, Kraken2 evaluates the taxonomic labels of all matching k-mers and determines a consensus using an LCA approach. This method ensures high sensitivity and specificity, even for closely related taxa.

- *Confidence scoring and filtering*: A minimum confidence threshold of 0.1 was applied to reduce false positives, as recommended for accurate sub-strain level discrimination in low-diversity datasets.

- *Output interpretation*: Kraken-generated reports were used to determine the proportion of reads classified at each taxonomic level (phylum to strain). Classification summaries were cross-validated against metadata from Probiotics Australia and known reference genomes.

Although Kraken2 enables fast and sensitive classification, we recognize its limitations in distinguishing extremely close strains or identifying novel variants. Therefore, while Kraken2 served as a reliable tool for initial taxonomic profiling, future studies will incorporate complementary methods such as average nucleotide identity (ANI) and multi-locus sequence typing (MLST) for deeper strain resolution and validation.

### Identification of Surface and Secreted Proteins

Bacterial protein sequences were analyzed to predict substrates cleaved by various signal peptidases and to determine their subcellular localization, which is essential for understanding protein–protein docking interactions. Protein sequences in FASTA format were processed using SignalP 6.0 (https://services.healthtech.dtu.dk/services/SignalP-6.0/) to identify signal peptides and predict cellular locations. SignalP 6.0 applies deep learning algorithms trained on extensive datasets of experimentally verified signal peptides, enhancing the accuracy of predictions. SignalP 6.0 classifies sequences into four categories: no signal peptide, Sec-type signal peptide (Sec-SP), Tat-type signal peptide (Tat-SP), and lipoprotein signal peptide (lipo). Sec-type signal peptides direct proteins to the Sec translocon pathway for secretion, whereas Tat-type signal peptides guide proteins through the twin-arginine translocation (Tat) pathway, typically for proteins that fold in the cytoplasm before export. Sec-type and lipoprotein signal peptides are typically associated with cell surface proteins in both Gram-positive and Gram-negative bacteria [29]. LipoP 1.0 was used to further refine the identification of lipoprotein substrates processed by SPase II, characterized by a lipobox motif. LipoP specifically detects four-residue lipobox motifs (e.g., [LVI][ASTG][GAS]C) at the cleavage site, which are essential for lipid modification and membrane anchoring of bacterial lipoproteins. Sequences suspected of being processed by SPase III, such as those controlling type IV pilin-like proteins, were manually analyzed for specific N-terminal leader sequences. These leader sequences, often rich in positively charged residues, play a critical role in type IV pilus assembly and bacterial adhesion. All predictions were cross-referenced with known data from UniProt and curated databases to confirm signal peptide type, cleavage site accuracy, and subcellular localization. Discrepancies between computational predictions and database annotations were carefully examined, and where necessary, multiple tools were used in parallel to improve prediction confidence.

### *Molecular Docking Analysis of Aβ*_*42*_* and Tau with Probiotic Proteins*

#### Tertiary Structure Identification

The surface and secreted proteins of probiotics were analyzed through molecular docking. The predicted 3D structures of Aβ_42_ and tau were retrieved from the Protein Data Bank (PDB) and AlphaFold databases. These structures were carefully selected based on high-resolution crystallographic or cryo-EM data when available, ensuring biologically relevant conformations. To obtain the 3D structures of probiotic proteins, a similarity search using the Basic Local Alignment Search Tool (BLAST) algorithm was performed against the PDB using RCSB-PDB (www.rcsb.org), identifying homologous proteins from AlphaFold structures [30, 31]. The BLAST search was optimized by adjusting parameters such as matrix type and gap penalties to maximize sensitivity while maintaining specificity in identifying homologous proteins. Only templates with an identity of ≥ 85% were selected [32]. This threshold was chosen to ensure structural conservation and functional relevance while minimizing errors due to distant homology modeling. The 3D structures were visualized using PyMOL [33], which allowed for detailed structural inspection, including secondary structure elements, active site geometry, and potential interaction sites. Key structural features, such as β-sheets in Aβ_42_ and phosphorylation-prone residues in tau, were examined to assess potential docking interactions.

#### Protein-Protein Docking Analysis

To predict the high-quality structures of Aβ_42_- and tau-probiotic protein complexes, molecular docking was performed using the ClusPro server v2.0 (https://cluspro.org). PDB files corresponding to tau, Aβ_42_, and probiotic proteins were submitted to the ClusPro 2.0 platform [34–36] for docking analysis, which predicted binding affinities and patterns. To ensure accuracy, structures were energy-minimized before submission to ClusPro to remove steric clashes and optimize side-chain conformations. ClusPro generates multiple conformations of ligand binding, selects the lowest energy configurations, and clusters the top 1000 conformations based on root-mean-square deviation (RMSD) differences. The top 10 clustering centers were manually examined to identify the most likely binding patterns. Key docking parameters, including electrostatic, van der Waals, and desolvation energy scores, were evaluated to rank docking conformations. The lowest energy conformations were chosen for further analysis of tau and Aβ_42_ proteins. Docked structures and interface residues were visualized and analyzed using PyMOL v2.5.4 [33], with detailed measurements of hydrogen bonds, salt bridges, and hydrophobic interactions to assess binding stability. Images were taken for proteins that docked with both tau and Aβ_42_.

#### Docking Specificity to Tau and Aβ_42_ Hotspots

This study aimed to investigate the specificity of probiotics interactions with tau and Aβ_42_ hotspots. The Docked complexes generated by ClusPro 2.0 were submitted to two bioinformatics tools, AGGRESCAN (http://bioinf.uab.es/aggrescan/) and PDBsum (http://www.ebi.ac.uk/thornton-srv/databases/pdbsum/), to assess hotspot interactions.

First, the AGGRESCAN server was employed to identify aggregation-prone regions (hotspots) within the tau and Aβ_42_ sequences. The FASTA sequences of tau and Aβ_42_ were submitted, and hotspots were determined based on aggregation tendencies, which were derived from intracellular aggregation assays conducted in living cells with intact protein quality control mechanisms [37, 38]. AGGRESCAN results were cross-referenced with existing literature on amyloidogenic regions of tau and Aβ_42_ to ensure biological relevance. AGGRESCAN can predict protein fragments that contribute to the aggregation of neurodegeneration-related proteins and assess the effects of genetic mutations on aggregation propensity [7]. Next, the PDBsum server [39] was used to submit docked complexes generated by ClusPro. The interactions of probiotic proteins with tau and Aβ_42_ hotspots were analyzed, comparing the interacting residues with the previously identified hotspots using AGGRESCAN. Structural interaction maps generated by PDBsum were analyzed to identify critical contact residues, solvent accessibility, and secondary structure contributions at the binding interface, enhancing the understanding of probiotic protein docking specificity.

### Probiotic Cell Lysis Protocol

Bacterial cells were harvested by centrifugation at 5000 × g for 10 min. The pellet was resuspended in B-PER Reagent (Thermofisher, Australia) containing lysozyme (2 µL/mL) and DNase I (2 µL/mL). EDTA-free protease inhibitors were added to prevent proteolysis. For every gram of cell pellet, 2 mL of B-PER II Reagent was added, and the suspension was pipetted up and down until homogeneous. The mixture was incubated at room temperature for 10–15 min to ensure efficient lysis. Finally, the lysate was centrifuged at 15,000 × g for 5 min to separate the soluble proteins in the supernatant from the insoluble pellet.

### *Probiotic Treatment and Inhibition of Aβ*_*42*_* Aggregation*

Aβ_42_ was synthesized by Genscript (Piscataway, New Jersey, USA) at > 95% purity and used without further purification. Ultrapure, metal-free MilliQ water (18 MΩ, Millipore) was used throughout for buffer preparation. A stock solution of thioflavin T (ThT) (Sigma-Aldrich, St. Louis, Missouri, USA) was prepared in metal-free water and filtered through a 0.22-µm membrane (Millipore). The effects of live probiotics (LPB), lysed probiotics (lPB), and probiotic growth supernatant (PBGS) on Aβ_42_ aggregation kinetics were assessed. Probiotic strains *Lactobacillus reuteri* (*L. reuteri*) 59 and 06, *Lactobacillus paracasei* (*L. paracasei*) 05, and *Lactobacillus rhamnosus* (*L. rhamnosus*) 12, or their derivatives, were mixed with monomeric Aβ_42_ peptide. A 50-µL aliquot of LPB, lPB, PBGS, or LPBP was incubated with 2 μL of 25 μM Aβ_42_ solution. Microplates were sealed and incubated at 37 °C for 46 h in a FLUOstar OMEGA (BMG Labtech, Ortenberg, Germany).

Aβ_42_ concentration was estimated by phenylalanine absorbance at 260 nm (ε = 195 M^−1^ cm^−1^ × n phenylalanine). Lyophilized peptides were initially dissolved in 1,1,1,3,3,3-hexafluoroisopropanol (HFIP) until fully solubilized. HFIP was then evaporated under a gentle nitrogen (N₂) stream, leaving a visible peptide film, which was resuspended in dimethylsulfoxide (DMSO).

Aβ_42_ peptide (20 µM) was prepared in 50 mM Tris buffer (pH 7.4) containing 5 µM ThT in a black-walled, clear-bottom, sterile 96-well microplate (BMG Labtech, Ortenberg, Germany), with a final volume of 200 µL. The plate was sealed with a transparent film and incubated at 37 °C in a FLUOstar OPTIMA microplate reader (BMG Labtech, Ortenberg, Germany) for up to 46 h. Fluorescence was recorded every 5 min using 440-nm excitation (30 flashes) and 485-nm emission, with an instrument gain of 1800. Plates were continuously shaken prior to each reading, with a minimum of two replicates per experiment. ThT fluorescence was measured from the bottom of the plate at all time points.

For imaging, 10 µL of fibril-containing samples from the ThT assay was applied to 300-mesh copper formvar-coated TEM grids (ProSciTech, Townsville, Australia). After 10 min of incubation, excess sample was wicked off with a Kimwipe, and grids were gently rinsed with water for 20 s before air drying overnight. Images were acquired using a Zeiss Merlin (ZEISS, Oberkochen, Germany) FEGSEM at 30-kV acceleration voltage. Data analysis and visualization were performed using GraphPad Prism 10.0.

### *PHF Tau Aggregation Inhibition Assay Using Probiotic Derivatives*

To evaluate the potential of probiotic-derived preparations to inhibit tau fibrillization, we employed a thioflavin T (ThT) fluorescence assay to monitor aggregation kinetics of the synthetic tau peptide PHF (sequence: GKVQIINKKLDL), a well-established aggregation-prone segment corresponding to residues 273–284 of tau. PHF273–284 peptide was synthesized to > 95% purity (Genscript, USA) and used without further purification.

Lyophilized PHF273–284 peptide was dissolved in ultrapure water and diluted in 10 mM HEPES buffer (Gibco) to a final concentration of 20 µM. Aggregation was induced by the addition of heparin (15–19 kDa, Sigma-Aldrich H3149) at a final concentration of 2 µM, a known enhancer of tau fibril formation. Thioflavin T (ThT; Sigma-Aldrich T3516) was added to a final concentration of 5 µM for fluorescence monitoring.

Test conditions included PHF273–284 peptide incubated alone, or co-incubated with either (i) live probiotic bacterial cultures (LPB), (ii) lysed probiotics (lPB), or (iii) probiotic bacterial growth supernatant (PBGS). Scrambled PHF peptide (sequence: DVLYSVQPKIKV) was used as a negative control. All test samples were prepared in triplicate.

The aggregation kinetics were monitored using a FLUOstar Omega microplate reader (BMG Labtech) over a 46-h period. Assays were performed in black 96-well clear-bottom plates, sealed with optical film to prevent evaporation. Fluorescence measurements were recorded at 15-min intervals with the following parameters: excitation at 440 nm, emission at 485 nm, and gain settings adjusted using ThT + heparin and ThT-alone control wells. Prior to each reading cycle, plates were shaken using a double orbital motion at 400 RPM for 15 s to ensure homogeneous sample mixing.

ThT fluorescence intensity (relative fluorescence units (RFU)) was plotted against time to assess the extent and kinetics of PHF fibril formation. Reduced fluorescence in treatment groups, compared to PHF-alone controls, was interpreted as evidence of fibrillization inhibition. All kinetic curves were analyzed using GraphPad Prism 10.0. Statistical comparisons were conducted using one-way ANOVA with Tukey’s post hoc test. Significance was set at *p* < 0.05.

### Probiotic Passage Through the Gastrointestinal Tract and Detection in Feces

In this study, probiotics were administered to healthy individuals over a 10-day period to assess their ability to traverse the gastrointestinal (GI) tract and determine their presence in fecal samples. The probiotic strains used included *L. reuteri* 59 and 06, *L. paracasei* 05, and *L. rhamnosus* 12, which were orally ingested. Fecal samples were collected at baseline (before administration) and at the end of the 10-day period.

Participants were randomly separated into two groups by gender to ensure balance and comparability between male and female participants. Eligibility screening was performed by a certified Clinical Psychologist at Korle-Bu Teaching Hospital. Inclusion criteria for healthy individuals included the following: (i) age between 30 and 50 years; (ii) abstention from consuming any dietary supplements, including probiotics, yogurts with live cultures, or immune-enhancing supplements; (iii) no current medications; and (iv) a Montreal Cognitive Assessment (MoCA) score above 26 during the study period.

Exclusion criteria included recent use of probiotics, antibiotics, anti-inflammatory drugs, gastrointestinal treatments within the past 3 months, or an inability to live independently. Of 30 individuals screened, 20 healthy participants were enrolled in the study.

### *Quantification of Lactobacillus reuteri in Feces*

Fecal samples were collected from participants both before and after probiotic consumption. Each participant was provided with a DNA/RNA Shield fecal collection tube containing a DNA-stabilizing preservative reagent and a ColOFF device (Zymo Research, California, USA). Participants were instructed to deposit fecal samples into the tubes and gently mix until the sample was fully submerged in the preservative. The collected samples were sealed and stored at room temperature (15–25 °C) before transportation to the laboratory, where they were immediately stored at − 80 °C for further analysis.

For genomic DNA isolation, fecal samples were initially homogenized with Precellys 24 tissue homogenizer (Thermofisher, Australia) using Cryolys with setting of 5000 × 20 × 3 at 4 °C followed with a gentle spin; then the FavorPrep Stool Isolation Mini Kit (Favorgen Biotech Corp, Taiwan) was used, as per the manufacturer’s instructions. DNA concentrations were measured using the Invitrogen Qubit dsDNA BR Assay Kit (Invitrogen, USA).

Finally, quantitative real-time PCR (qPCR) was used to quantify the presence of *L. reuteri* DNA in fecal samples, utilizing the QuantStudio™ 7 Pro Real-Time PCR system (Thermo Fisher Scientific). Specific primers targeting *L. reuteri* were employed for accurate detection, including *L. Reuteri*-F (GAAGATCAGTCGCAYTGGCCCAA) and *L. Reuteri*-R (TCCATTGTGGCCGATCAG), along with general bacterial primers, General bacteria-F (ACTCCTACGGGAGGCAGCAG) and General bacteria-R (ATTACCGCGGCTGCTGG) [40]. The qPCR assays were conducted in 10-μL reaction volumes using 5μL PowerTrack SYBR Green qPCR Master Mix (Thermo Fisher Scientific), 10 μM forward and reverse primers, and 30 ng of extracted DNA under the following conditions: an initial denaturation step at 95 °C for 20 s, followed by 40 cycles of 95 °C for 15 s, 60 °C for 1 min, with a final extension at 72 °C for 5 min. Melting curve analysis was performed post-amplification to confirm the specificity of the PCR products. All samples were analyzed in duplicate, and DNA levels were normalized to universal bacteria primer and expressed relative to the level of *L. reuteri* before supplementation using the Pfaffl method (PMID11328886). The sequences and efficiency of qPCR primers are designed and evaluated in accordance with the MIQE PCR guidelines (PMID19246619) and are shown in Supplementary Table 1.

### Statistical Analysis

Statistical analysis was performed using one-way ANOVA followed by Dunnett’s post hoc test (GraphPad Prism version 7.00). The analysis was used to identify general trends and guide descriptive comparisons across groups. *p*-values are reported in the tables or figures to emphasize formal statistical significance.

## Results

### Probiotics Genome Assembly and Annotation Results

Following quality control using Trimmomatic software, reads containing adapter sequences and those with more than 5% unknown nucleotides were removed. Additionally, low-quality reads with over 20% of bases having a *Q*-value ≤ 10 were eliminated to ensure high data integrity. After trimming, all remaining reads showed high quality, with no adapters detected.

The de novo genome assembly, conducted using the Unicycler pipeline, resulted in genome sizes ranging from 1,967,015 to 3,381,728 base pairs and the number of contigs for the assembled genomes varied between 31 and 192, with a mean of 88.3, median of 73, and standard deviation of 46.4 across the probiotic species analyzed (Table 1). The Prokka pipeline was employed for genome annotation, revealing coding sequences (CDS) ranging from 1891 to 3195 (Table 1).

**Table 1:** Summary of bacterial genome size, contig distribution, and functional elements identified using the Prokka pipeline analysis: an overview of the genomic characteristics of various probiotic bacterial strains, including genome size, number of contigs, and functional elements such as coding sequences (CDS), ribosomal RNA (rRNA), transfer RNA (tRNA), and transfer-messenger RNA (tmRNA)

Bacteria	Genome size (base pairs)	Contigs	CDS	rRNA	tRNA	tmRNA
*L. paracasei 05*	3,050,771	110	2873	2	47	1
*L. reuteri 06*	2,008,334	192	1970	2	32	1
*L. helveticus 07*	1,967,015	181	1988	3	56	1
*L. rhamnosus 12*	2,929,959	72	2743	4	47	1
*B. longum 24*	2,309,682	38	1891	3	56	1
*L. plantarum 50*	3,381,728	73	3210	3	58	1
*L. plantarum 51*	3,347,682	60	3190	3	58	1
*L. plantarum 52*	3,207,724	38	3008	2	29	1
*L. plantarum 53*	3,313,810	83	3097	2	62	1
*L. plantarum 54*	3,373,057	97	3195	3	56	1
*L. rhamnosus 56*	2,814,671	72	2626	4	2	1
*L. helveticus 58*	1,998,072	186	2029	3	56	1
*L. reuteri 59*	1,982,807	119	1927	2	59	1
*L. rhamnosus 62*	2,941,364	31	2730	2	47	1
*L. plantarum 63*	3,296,709	41	3101	3	59	1
*L. paracasei 64*	3,048,905	115	2868	2	47	1

Each probiotic strain exhibited a single mRNA, and the total number of identified proteins per strain ranged from 1897 to 3213, and a 35.91 to 41.84% proportion was deemed hypothetical (Table 2). Notably, *L. plantarum* 51 had the highest number of hypothetical proteins (1350), while *L. reuteri* showed the lowest number (692) (Table 2).

**Table 2:** Bacterial protein count, hypothetical protein prevalence, and SignalP/Tat predictions of protein localization: the total number of proteins, the proportion of hypothetical proteins, and the SignalP/Tat predictions for protein localization across different bacterial strains. Hypothetical proteins are those predicted to be expressed based on the genome sequence but lack experimental confirmation. Localization predictions include lipoproteins (Lipo), pilins (Pilin), signal peptides (Sp), Tat pathway proteins (Tat), cytoplasmic proteins, and secreted proteins

Bacterial ID	Protein number	Hypothetical proteins	% hypothetical proteins	Lipo	Pilin	Sp	Tat	Cytoplasmic proteins	Secreted proteins
*L. paracasei 05*	2873	1170	40.72	56	3	74		2740	133
*L. reuteri 06*	1970	758	38.48	14	3	30		1923	47
*L. helveticus 07*	1988	813	40.90	36	2	63	1	1886	102
*L. rhamnosus 12*	2743	1101	40.14	48	2	87		2606	137
*B. longum 24*	1897	785	41.38	46		43		1808	89
*L. plantarum 50*	3213	1350	42.02	59	4	96		3054	159
*L. plantarum 51*	3193	1336	41.84	59	4	95		3035	158
*L. plantarum 52*	3011	1207	40.09	98	4	57		2852	159
*L. plantarum 53*	3100	1248	40.26	58	4	97		2941	159
*L. plantarum 54*	3198	1325	41.43	55	4	94		3045	153
*L. rhamnosus 56*	2626	1054	40.14	47	2	74		2503	123
*L. helveticus 58*	2029	836	41.20	36	2	44	1	1946	83
*L. reuteri 59*	1927	692	35.91	15	3	42		1867	60
*L. rhamnosus 62*	2730	1089	39.89	51	2	75		2602	128
*L. plantarum 63*	3104	1265	40.75	58	4	100		2942	162
*L. paracasei 64*	2868	1166	40.66	57	3	74		2734	134

### Identification and Characterization of Surface/Secreted Proteins in Probiotic Strains

The subcellular localization of proteins plays a crucial role in determining their interactions, as the surrounding environment can significantly influence the accessibility and structural conformation of binding sites. Most of the identified proteins were localized within the bacterial cytoplasm (≈ 95.42%), while a smaller subset (≈ 4.58%) contained signal peptides associated with secretion, which are vital for the extracellular release of proteins (Table 2).

A total of 23 surface/secreted proteins out of 1988 derived from the probiotic strains were identified based on their signal peptides and potential roles in mediating interactions with host or pathogenic bacterial proteins (Table 2). These proteins were selected for further downstream analysis, given their hypothesized involvement in host-microbe interactions and their potential therapeutic relevance. These proteins were selected for further downstream analysis based on the presence of predicted signal peptides, lipobox motifs, and non-cytoplasmic localization, as determined by SignalP 6.0 and LipoP 1.0. Only proteins classified as secreted, membrane-bound, or surface-associated were retained for docking, while cytoplasmic proteins were excluded to ensure relevance to host–microbe interactions.

### *Protein–Protein Docking Between Tau and Aβ*_*42*_* with Probiotic Proteins*

Initially, we retrieved the 3D structures of Aβ_42_ and tau from the Protein Data Bank (PDB) and AlphaFold database with accession numbers 1iyt and AF-P10636, respectively. For the 3D structure determination of probiotic proteins, the BLAST algorithm was used. Our findings revealed that the predicted probiotic proteins exhibited high identity to known proteins, ranging from 83.1 to 100% (Table 3). Only proteins with ≥ 85% sequence identity were considered acceptable for inclusion in docking studies to ensure structural accuracy and relevance.

**Table 3:** Homology search results for probiotic proteins against biomolecular databases: the results of homology searches conducted for selected proteins from various probiotic strains against key biomolecular databases. It includes the number of proteins identified with significant homology

Bacteria	Protein number	Align no	Protein name	Organism	Length	Score (bits)	Identities
***L. paracasei 05***	1647	1	Serine-type d-Ala-d-Ala carboxypeptidase	*Lactobacillus paracasei*	436	846.7	100
	151	1	ABC transporter substrate-binding protein/permease	*Lacticaseibacillus paracasei* subsp. *tolerans*	486	944.5	100
***L. reuteri 06***	473	1	ABC transporter substrate-binding protein/permease	*Lactobacillus reuteri*	487	957.2	100
	1908	1	Beta-lactamase	*Lactobacillus reuteri*	414	816.6	100
***L. helveticus 07***	1533	1	d-Alanyl-d-alanine carboxypeptidase	*Actobacillus helveticus*	660	1335.5	100
	487	1	ABC transporter permease	*Lactobacillus helveticus*	494	944.9	100
***L. rhamnosus 12***	1983	1	d-Alanyl-d-alanine carboxypeptidase	*Lactobacillus rhamnosus* (strain ATCC 53103/GG)	436	850.5	100
	1295	1	Amino acid ABC transporter amino-acid-binding/permease protein	*Lactobacillus rhamnosus*	482	922.5	100
***B. longum 24***	1397	1	GH43_C domain-containing protein	*Bifidobacterium longum*	531	1106.7	99.8
	480	1	Calcineurin-like phosphoesterase	*Bifidobacterium longum* E18	604	1185.6	100
***L. plantarum 51***	2297	1	Maltodextrin-binding protein	*Actiplantibacillus plantarum*	417	836.6	100
	258	1	Nopaline-binding periplasmic protein	*Lactiplantibacillus plantarum* subsp.* plantarum*	478	934.9	100
***L. plantarum 53***	2650	1	ABC transporter permease	*Lactiplantibacillus paraplantarum*	491	956.8	100
	1343	1	Phosphate-binding protein	*Lactiplantibacillus plantarum*	300	584.3	100
***L. plantarum 54***	3220	1	DUF1541 domain-containing protein	*Lactiplantibacillus plantarum*	185	196.1	55.1
***L. rhamnosus 56***	2382	1	Membrane protein insertase YidC 1	*Lactobacillus rhamnosus*	330	654.1	100
	331	1	PII-type proteinase	*Lactobacillus paracasei*	1902	2885.9	75.1
***L. reuteri 59***	168	1	ABC transporter permease	*Lactobacillus* sp. UMNPBX18	503	972.6	100
***L. rhamnosus 62***	1955	1	ABC transporter permease subunit	*Lacticaseibacillus rhamnosus*	486	945.7	100
	2376	1	Glycine betaine/carnitine/choline-binding protein	*Lactobacillus rhamnosus*	309	623.6	100
***L. plantarum 63***	1172	1	Membrane protein insertase YidC	*Lactobacillus plantarum* subsp.* plantarum*	277	542.7	100
***L. paracasei 64***	458	1	Glutamine-binding periplasmic protein	*Lactobacillus paracasei*	488	946	100

Protein–protein docking analysis, performed using the ClusPro server, showed a range of binding energies between tau and probiotic proteins (Table 4). The lowest energy values for tau interactions ranged from − 960.4 to − 1922.4, with protein 2462 (Maltodextrin-binding protein MdxE) from *L. plantarum 51* showing the least affinity and protein 2650 (Membrane-bound lytic murein transglycosylase F) from *L. plantarum 53* showing the highest affinity. Similarly, docking with Aβ_42_ revealed energies ranging from − 713 to − 1439.4, where protein 1647 from *B. longum 24* had the lowest affinity, and protein 151 from *L. paracasei 05* showed the highest affinity (Table 4).

**Table 4:** Molecular docking energy scores indicating high affinity binding of selected bacterial proteins to Tau and Aβ_42_ aggregation sites: lowest energy scores for docking interactions between selected bacterial proteins and tau and Aβ_42_. The lower the score, the higher the binding affinity, indicating a potential inhibitory effect on tau and Aβ_42_ aggregation, which are key features in Alzheimer’s pathology

Bacteria	Protein ID	Tau	Aβ_42_
***L. paracasei* 05**	151	− 1500.9	− 1439.4
	1647	− 1199.2	− 713
***L. reuteri* 06**	473	− 1416.3	− 1204
	1908	− 1540.6	− 1311.5
***L. helveticus* 07**	485	− 1520.1	− 1245.1
	1533	− 1193.6	− 922.8
***L. rhamnosus* 12**	1295	− 1723.3	− 1307.8
	1983	− 1256.3	− 755
***B. longum* 24**	480	− 1326.4	− 992
	1397	− 1127.3	− 1032.4
***L. plantarum* 50**	1789	− 1818.6	− 741.1
	2462	− 960.4	− 845.8
***L. plantarum 51***	2297	− 960.4	− 845.8
	258	− 1408.1	− 1217.4
***L. plantarum* 52**	1139	− 1408.1	− 1217.4
***L. plantarum* 53**	1343	− 1035.7	− 815.9
	2650	− 1818.6	− 1286.5
***L. plantarum* 54**	3220	− 1221.3	− 1106.1
***L. rhamnosus 56***	331	− 1147.5	− 878.2
	2382	− 1725.5	− 1356.1
***L. helveticus 58***	388	− 1520.1	− 1245.1
***L. reuteri* 59**	168	− 1613.3	− 1430.3
	352	− 1416.3	− 1204
***L. rhamnosus* 62**	2376	− 1076.2	− 883.6
	1955	− 1922.4	− 1340.1
***L. plantarum 63***	1400	− 1818.6	− 1286.5
	1172	− 1611.3	− 1137.2
***L. paracasei* 64**	458	− 1767.8	− 1280
	1662	− 1199.2	− 713

Among the surface and secretory probiotic proteins analyzed, strains such as *L. reuteri* 59 and 06, *L. paracasei* 05, and *L. rhamnosus* 12 exhibited consistently strong binding affinities to both tau protein and Aβ_42_ peptide (Table 4). These strains showed strong docking scores across multiple proteins, with *L. reuteri* 06 and 59 consistently interacting with both tau and Aβ_42_. *L. paracasei* 05 exhibited particularly high affinity for Aβ_42_, while *L. rhamnosus* 12 had the highest affinity for tau, indicating a strong potential for inhibiting the aggregation of both proteins. Conversely, strains such as *L. helveticus* 07 and 58 and *B. longum* 24 displayed lower and less consistent affinities (Table 4).

**Fig. 1:**
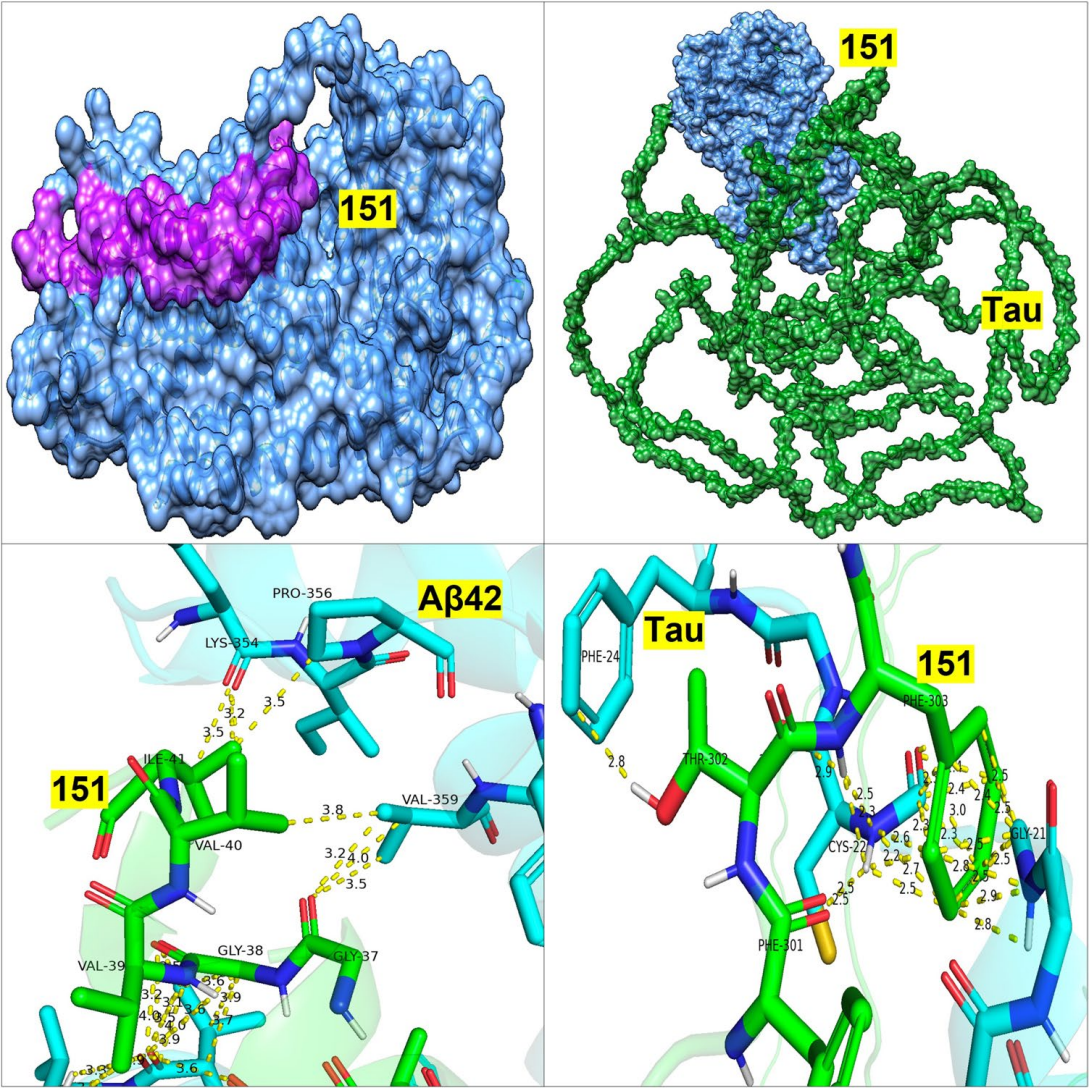
PA05—docking of PA 5 Protein 151 with Aβ_42_ and Tau

**Fig. 2:**
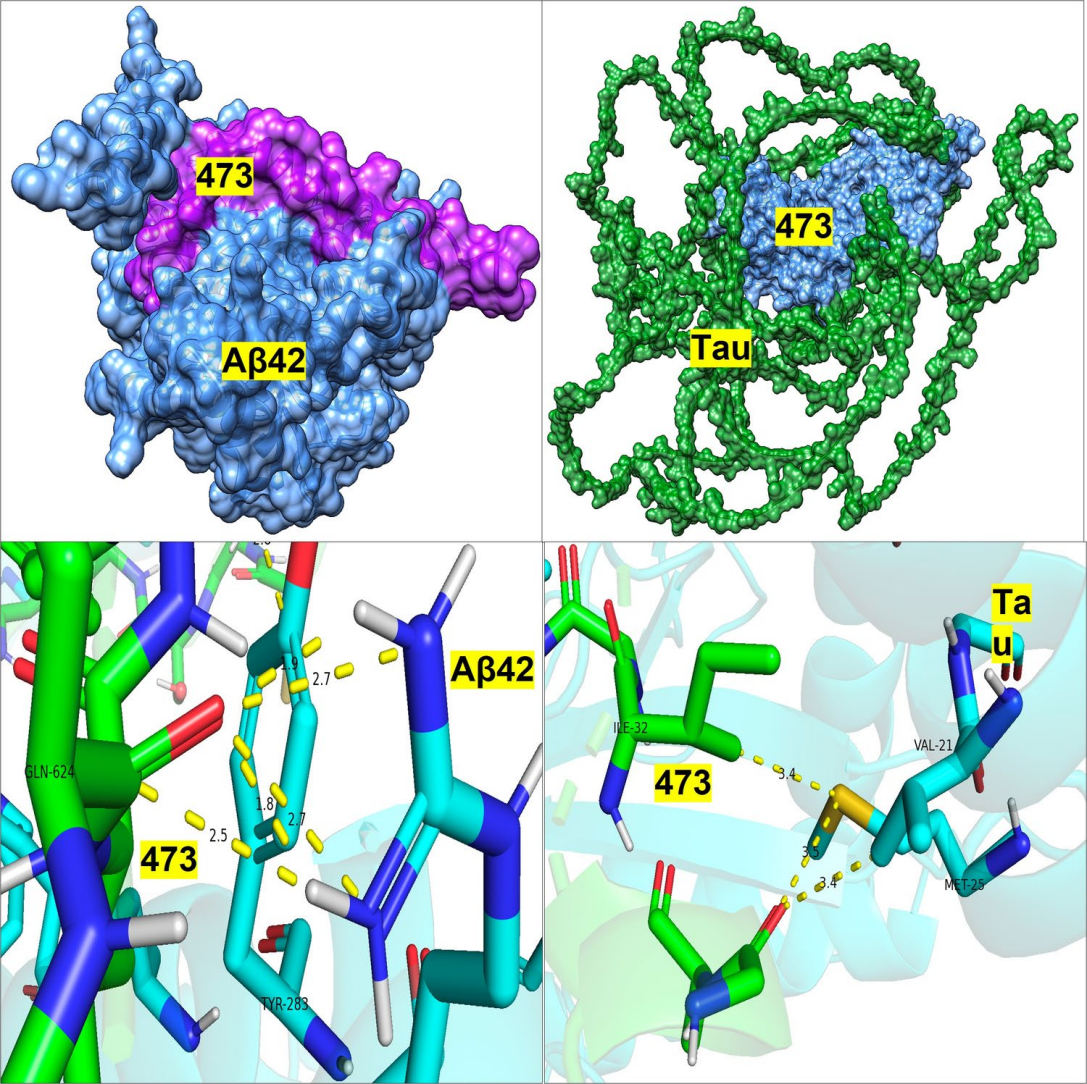
PA06—docking of PA 6 Protein 473 with Aβ_42_ and Tau

**Fig. 3:**
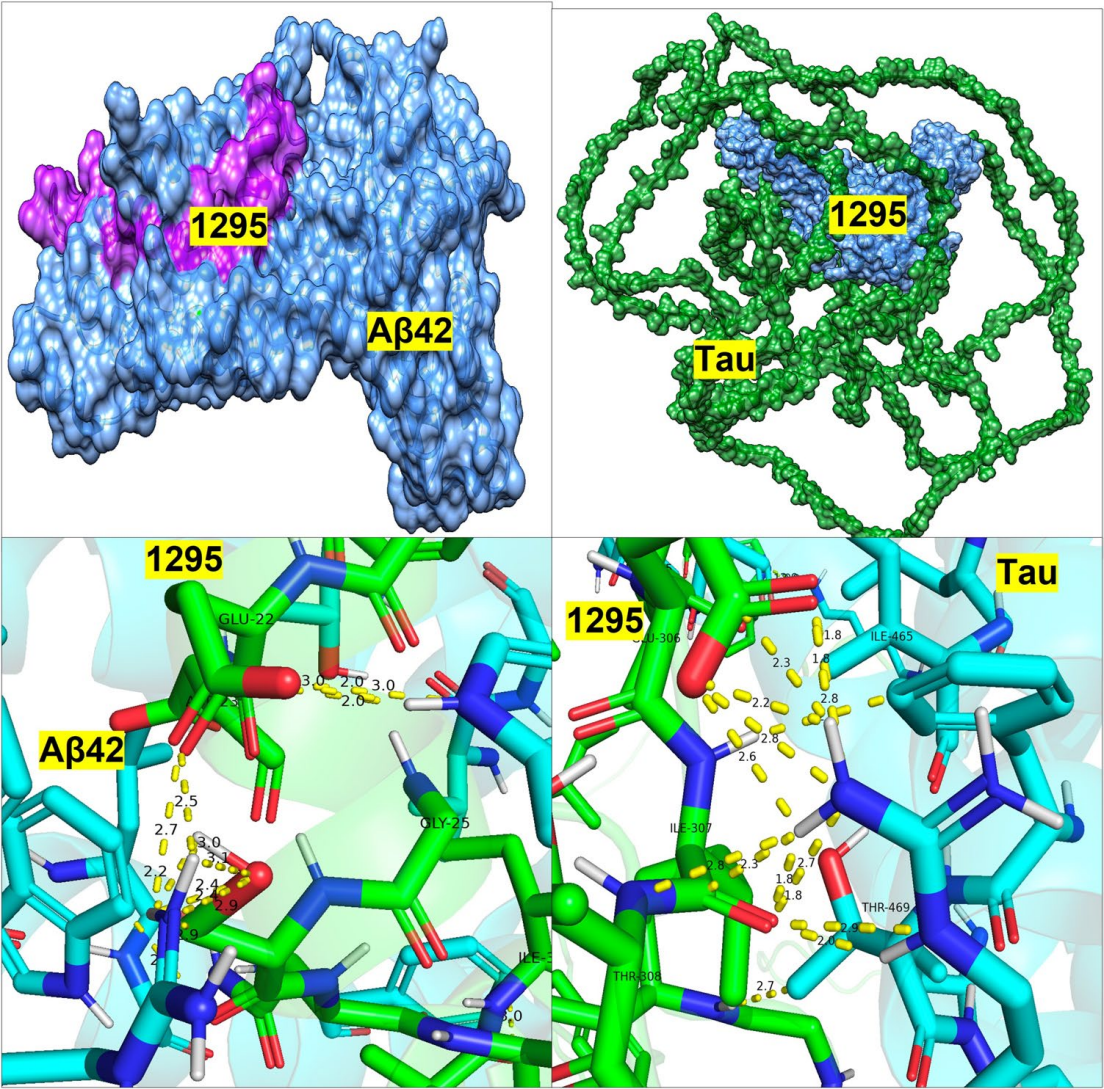
PA12—docking of PA 12 Protein 1295 with Aβ_42_ and Tau

**Fig. 4:**
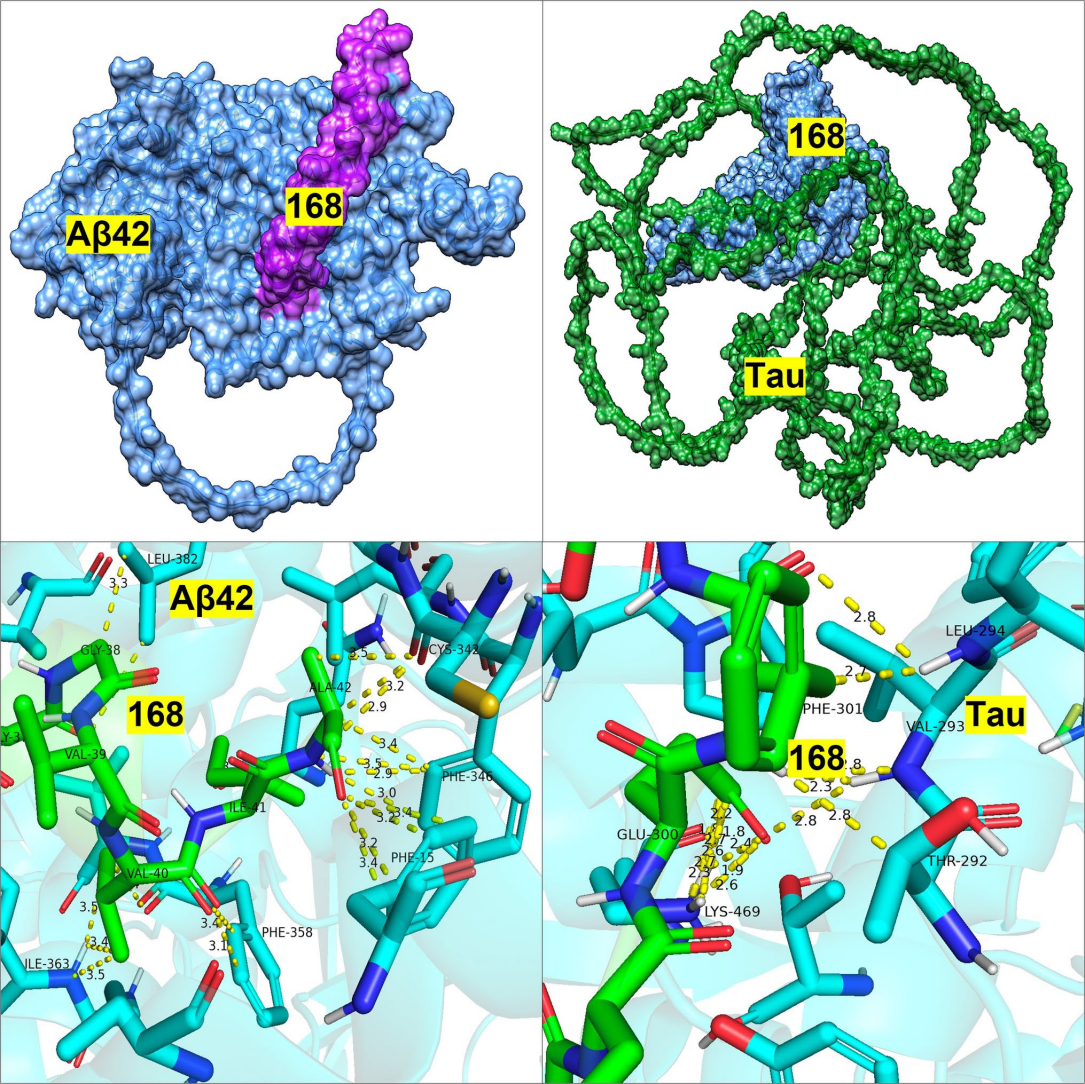
PA59—docking of PA 59 Protein 168 with Aβ_42_ and Tau

Further analysis of the binding sites between tau/Aβ_42_ and the selected probiotic proteins from *L. reuteri* 05 and 59, *L. paracasei* 05, and *L. rhamnosus* 12 was conducted using PDBsum (Table 5). Protein 168 (ABC transporter permease) from *L. reuteri* 59 exhibited a high number of non-hydrogen contacts with tau (337) and Aβ_42_ (146), along with strong docking scores. While the number of contacts alone does not always predict binding affinity, in this case, the high contact density coincided with low-energy docking conformations, suggesting enhanced interface stability and binding potential. This supports the likelihood of meaningful physical interaction and possible inhibitory activity. Protein ABC transporter substrate-binding protein/permease and beta-lactamase from *L. reuteri 06* also displayed notable interactions with tau (218 non-hydrogen contacts) and Aβ_42_ (109 non-hydrogen contacts) and tau (251 non-hydrogen contacts) and Aβ_42_ (178 non-hydrogen contacts). Protein Serine-type d-Ala-d-Ala carboxypeptidase from *L. paracasei* 05 similarly showed robust interactions, with tau (288 non-hydrogen contacts) and Aβ_42_ (51 non-hydrogen contacts). Finally, protein d-alanyl-d-alanine carboxypeptidase from *L. rhamnosus 12* demonstrated balanced interaction profiles with tau and Aβ_42_, making these strains strong candidates for further investigation (Figs. 1, 2, 3, and 4).

**Table 5:** PDBsum analysis of protein–ligand interactions: quantifying key contacts between bacterial proteins and Alzheimer’s disease peptides: the interactions between the four bacterial proteins and tau or Aβ_42_ as analyzed through PDBsum. Interactions include salt bridges, hydrogen bonds, and non-hydrogen contacts

		**Salt bridges**	**Hydrogen bonds**	**Non-hydrogen contact**
***L. paracasei 05***	1647 and tau	9	21	288
	1647 and Aβ_42_		3	51
	151 and tau	2	17	223
	151 and Aβ_42_	1	9	164
***L. reuteri 06***	473 and tau	6	22	218
	473 and Aβ_42_	1	3	109
	1908 and tau	1	19	251
	1908 and Aβ_42_	4	11	178
***L. helveticus 07***	1533 and tau	4	26	262
	1533 and Aβ_42_	3	13	133
	487 and tau	5	12	202
	485 and Aβ_42_	3	5	135
***L. rhamnosus 12***	1983 and tau	6	14	145
	1983 and Aβ_42_			37
	1295 and tau	3	9	149
	1295 and Aβ_42_	4	11	154
***L. reuteri 59***	168 and tau	2	9	337
	168 and Aβ_42_	3	6	146

Finally, to explore probiotic proteins interactions with aggregation-prone regions (hotspots) of tau and Aβ_42_, we utilized the AGGRESCAN server. Comparative analysis of binding residues revealed significant interactions between the probiotic proteins and the hotspots in tau and Aβ_42_. Proteins from *L. reuteri* 59 and 06 interacted with key residues in tau (303, 305–308, 624–628) and Aβ_42_ (20, 32, 34–42), while *L. paracasei 05* and *L. rhamnosus* 12 also showed interactions with critical aggregation-prone sites (Table 6), highlighting their potential to inhibit protein aggregation in AD.

**Table 6:** Identification of key hotspot residues on Tau and Aβ_42_ for high-affinity binding by probiotic-derived proteins: the hotspot residues in tau and Aβ_42_ that interact with selected bacterial proteins. Hotspot residues are amino acids within tau or Aβ_42_ that form significant binding contacts with bacterial proteins, influencing the protein–protein interaction and potentially altering the pathological aggregation process

Bacteria	Protein ID	Tau/Aβ_42_	Hotspot residue
*L. paracasei 05*	151	Aβ_42_	LEU 17, 34, PHE 20, ALA 21, ILE 31, 41, MET 35, GLY 37, 38, VAL 39, 40
	1647	Tau	VAL 623, 626, TYR 627, GLN 624, ILE 625
		Aβ_42_	PHE 20, ALA 21, LEU 34, MET 35, GLY 38
*L. reuteri 06*	473	Tau	PHE 303, VAL 305, GLU 306, ILE 307, THR 308, GLN 624, LYS 628
		Aβ_42_	PHS 20, ILE 32,34, MET 35, GLY 37, ILE 41, ALA 42
	1908	Tau	GLU 300, GLN 624, ILE 625, VAL 626, TYR 627, 628, GLU 300
		Aβ_42_	PHE 19,20, ALA 30, ILE 31, GLY 33, LEU 34, VAL 36, 39, 40, ILE 41
*L. rhamnosus 12*	1295	Tau	GLU 300, 306, ILE 307, PHE 301, 303, VAL 305, 623, ILE 307, THR 627, 308
		Aβ_42_	GLU 22, VAL 18, PHE 19, 20, ALA 21, ASP 23, ALA 30, 42, ILE 31, GLY 33, 38, LEU 34, 41
	1983	Tau	GLU 300, 306, PHE 301, THR 302, PHE 303, GLU 300, 306, VAL 623, ILE 625
		Aβ_42_	LEU 17, 34, PHE 20, MET 35, GLY 38, ILE 41, ALA 42
*L. reuteri 59*	168	Tau	GLU 300, PHE 301, THR 302, PHE 303, HIS 304, VAL 305, ILE 307, 625, 625, TYR 627
		Aβ_42_	LEU 17, PHE 20, ILE 32, 34, 41, MET 35, GLY 37, ALA 42
	352	Tau	PHE 303, VAL 305, 626, GLU 306, ILE 307, THR 308, GLN 624, ILE 625, TYR 627, LYS 628
		Aβ_42_	LEU 17, PHE 20, ILE 32, LEU 34, MET 35, GLY 37, ILE 41, ALA 42

### *Inhibition of Aβ*_*42*_* and Tau Aggregation and Fibril Formation by Probiotic Treatment*

**Fig. 5:**
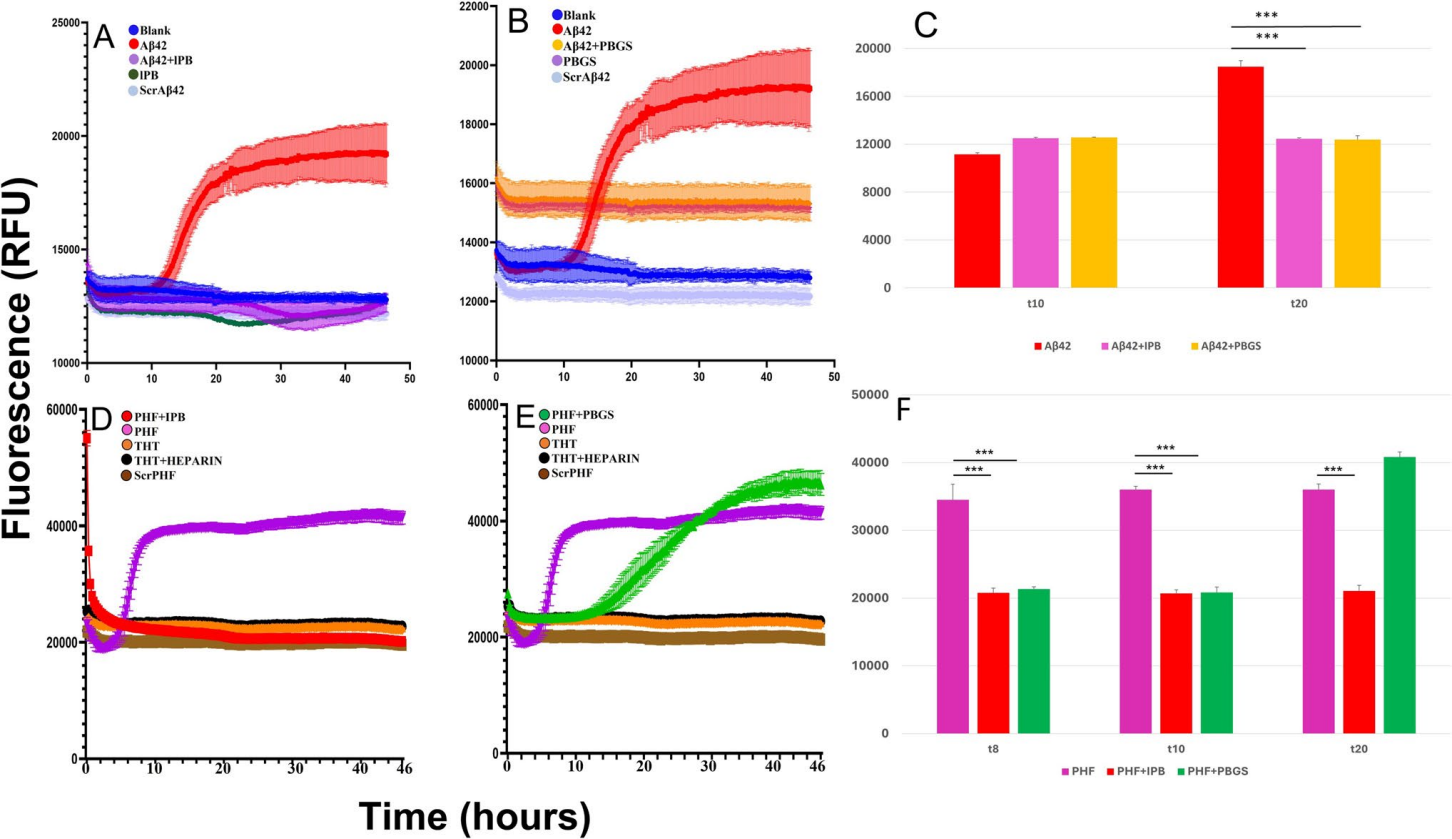
Effect of Lactobacilli probiotic strains on Aβ_42_ and Tau aggregation monitored by thioflavin T fluorescence assay. Thioflavin T (ThT) fluorescence kinetic assay was used to assess aggregation in the presence of live probiotics (LPB), lysed probiotics (lPB), and probiotics’ growth supernatant (PBGS). Fluorescence intensity (RFU) is plotted against time (hours), with higher fluorescence indicating greater aggregation. **A** Aβ_42_-alone condition (red curve) shows a strong increase in fluorescence. IPB co-incubated with Aβ_42_ (purple curve) shows significant reduction in fluorescence (*p* < 0.05). IPB alone (dark green) did not contribute to fluorescence. **B** Aβ_42_ alone (red curve) shows the characteristic increase in fluorescence. PBGS co-incubated with Aβ_42_ (orange curve) led to a reduction in fluorescence (*p* < 0.05), though to a lesser extent than IPB. PBGS alone (brown curve) did not display significant fluorescence changes. Both the blank control and the scrambled Aβ_42_ control remained stable, showing minimal fluorescence. **C** Quantification of fluorescence intensity at 10 h (t10) and 20 h (t20) for Aβ_42_ alone, Aβ_42_ + IPB, and Aβ_42_ + PBGS. ***p < 0.001 for comparisons between Aβ_42_ and both treated groups at t20, indicating significant inhibition of fibrillization. **D** PHF- (Tau peptide) alone condition (purple curve) shows a strong increase in fluorescence. IPB co-incubated with Aβ_42_ (red curve) shows significant reduction in fluorescence (*p* < 0.05). IPB alone (dark green) did not contribute to fluorescence. **E** PHF alone (purple curve) shows the characteristic increase in fluorescence. PBGS co-incubated with PHF (green curve) led to an initial reduction in fluorescence (*p* < 0.05), followed by significant fluorescence increase of fluorescence. PBGS alone (brown curve) did not display significant fluorescence changes. Both the blank control and the scrambled PHF control remained stable, showing minimal fluorescence. **F** Quantification of fluorescence intensity at 8, 10, and 20 h (t8, t10, t20) for PHF alone, PHF + IPB, and PHF + PBGS. ***p < 0.001 for all comparisons between PHF and peptide-inhibited conditions, demonstrating statistically significant suppression of PHF aggregation. Data represent mean ± SEM (*n* = 3 independent experiments). Statistical significance was assessed using one-way ANOVA with Tukey’s multiple comparisons test. All relevant *p*-values and statistical details are provided in the main text and highlighted in grey

In this study, we evaluated the impact of various probiotic strains and their derivatives on the aggregation of Aβ_42_ and a scrambled version of Aβ_42_ (scrAβ_42_) as well as a paired helical filament (PHF) tau peptide sequence GKVQIINKKLDL (PHF273-284) and a scrambled version sequence DVLYSVQPKIKV (scrPHF). The effects of live probiotics (LPB), lysed probiotics (lPB), and probiotics’ growth supernatant (PBGS) on Aβ_42_ and tau aggregation kinetics were assessed through thioflavin T (ThT) time-resolved fluorescence assays and transmission electron microscopy (TEM). All experiments were performed in triplicates and compared with their respective controls. Aggregation of Aβ_42_ followed the typical amyloidogenic profile that includes a long lag phase (10 h under the conditions of this experiment), and elongation and plateau phases (Fig. 1). The ThT fluorescence assay demonstrated significant inhibition of Aβ_42_ aggregation by probiotics and their derivatives (Fig. 5). The ThT fluorescence intensity, indicative of amyloid fibril formation, was markedly lower in samples treated with probiotics and their derivatives, confirming their inhibitory effect on Aβ_42_ aggregation (*p* < 0.05). Notably, lPB demonstrated superior efficacy in inhibiting the aggregation kinetics, effectively preventing the formation of both Aβ_42_ fibrils and aggregates (*p* < 0.05) (Fig. 5A, C). Furthermore, PBGS significantly inhibited Aβ_42_ fibrils (Fig. 5B, C); however, absence of the lag phase suggests the presence of potent fibrillization components in the culture bacterial media (Fig. 5B). PHF-tau fibrillization followed a similar trend to Aβ_42_ and was also inhibited following addition of IPB (Fig. 5D, F); however, PBGS inhibited PHF fibrilization in the initial phase but failed to stop aggression at 15 h reaching PHF levels at around 30 h (Fig. 5E).

Interestingly, the fluorescence intensity in the Aβ_42_/PHF + PBGS condition was notably elevated compared to Aβ_42_ alone and PHF alone overtime. This suggests that components within the probiotic growth supernatant (PBGS) may induce rapid β-sheet formation or structural rearrangement of Aβ_42_ and PHF, resulting in ThT binding. Such an effect may be attributed to the presence of bacterial-derived metabolites, peptides, or extracellular vesicles that act as potent aggregation-promoting agents or modulate ThT fluorescence directly. These factors may bypass the typical nucleation phase, leading to an absence (Aβ) or delay (PHF) of the elongation lag typically seen in amyloid fibrillization. While speculative, this observation points to the potential existence of amyloid-seeding or -accelerating activity within PBGS.

**Fig. 6:**
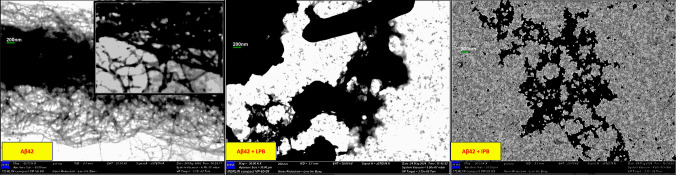
Inhibition of Aβ_42_ fibril formation by live and lysed probiotics visualized via TEM. Transmission electron microscopy (TEM) analysis of Aβ_42_ fibril formation in the absence or presence of live or lysed probiotics. Aβ_42_ monomeric peptides were incubated alone (left panel), with live probiotics (LPB; middle panel), or with lysed probiotics (IPB; right panel), including strains *Lactobacillus reuteri* 06 and 59, *Lactobacillus paracasei 05*, and *Lactobacillus rhamnosus 12*. The control (Aβ_42_) shows dense, mature fibrillar networks, indicative of amyloid aggregation. In contrast, samples treated with LPB exhibit visibly reduced and disrupted fibril structures. IPB-treated samples display further inhibition of fibril assembly, appearing mostly as small, dispersed aggregates or amorphous structures. These findings support ThT fluorescence results, indicating that both live and lysed probiotic treatments effectively impede Aβ_42_ fibrillogenesis. Images captured using Zeiss Merlin FEGSEM at 30 kV and 50,000–65,930 × magnification. Scale bars, 200 nm

TEM imaging further supported the ThT fluorescence findings. Electron microscopy images revealed a substantial reduction in the formation of Aβ_42_ fibrils in the presence of probiotics and their derivatives (Fig. 6). In comparison to the control, where extensive fibril networks were observed, samples treated with probiotics and their derivatives exhibited fewer and shorter fibrils, indicating effective inhibition of fibril formation (Fig. 6).

### Detection of Probiotics in Fecal Samples Following Oral Administration

**Fig. 7:**
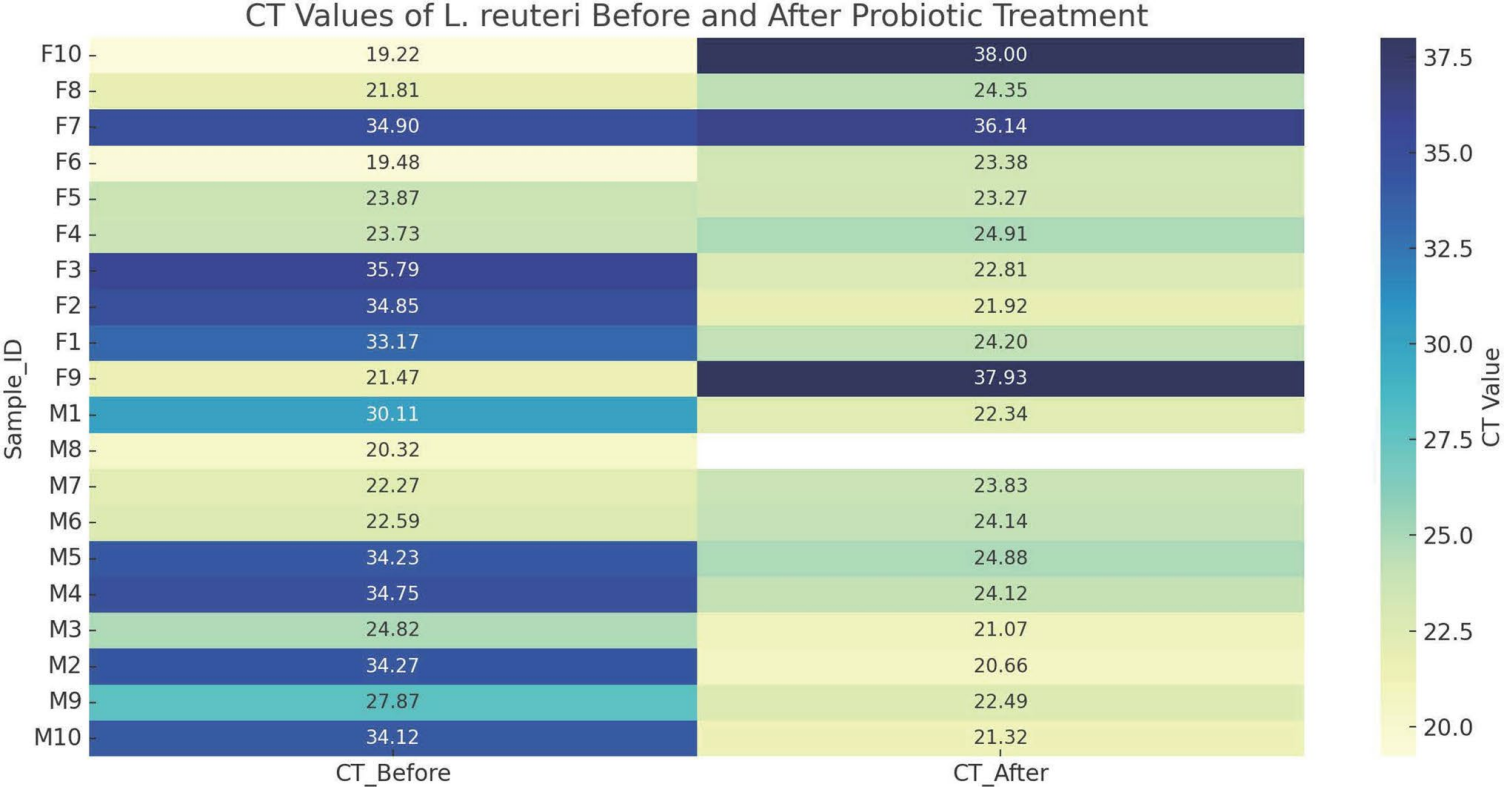
Heatmap of qPCR CT values for *Lactobacillus reuteri* before and after probiotic treatment. This heatmap illustrates the cycle threshold (CT) values obtained by quantitative PCR (qPCR) for *Lactobacillus reuteri* (LR) detection in stool samples of 20 participants (10 males, **M,** and 10 females, **F**), both prior to and 10 days following daily oral probiotic administration. Each row represents an individual, with CT values plotted for pre-treatment (left) and post-treatment (right) timepoints. Darker blue shades correspond to lower CT values, indicating higher bacterial abundance. Participants are grouped by sex and sorted within each group

To assess the presence and persistence of *L. reuteri* in the gastrointestinal tract of healthy individuals, we administered the probiotic strain orally for a period of 10 days and subsequently analyzed fecal samples. Using quantitative real-time PCR (qPCR), we quantified *L. reuteri* DNA in the fecal samples collected before and after the probiotic administration period (Fig. 7 and Supplementary Table 1). The proportion of LR-positive individuals rose from 10 out of 20 at baseline to 16 out of 20 post-treatment, indicating successful and rapid colonization in the majority of participants (Supplementary Table 1).

Quantitative PCR (qPCR) analysis demonstrated dramatic fold increases in LR abundance among those who were LR-negative at baseline (Fig. 7). Notable cases included participant M2, who showed a 13,593-fold increase; M10, with a 6170-fold increase; F3, with a 9848-fold increase; and F2, with an 8421-fold increase. Conversely, individuals who were already colonized with LR before treatment, such as M6, M7, F5, and F6, exhibited minimal fold changes (all < 1.5-fold), suggesting stable levels or a plateau effect in bacterial abundance.

The cycle threshold (CT) values, which are inversely related to target DNA abundance, shifted markedly from high values (CT 30–35) to lower values (CT 20–25) in newly colonized individuals, confirming a substantial increase in LR levels (Fig. 7). In contrast, the CT values for UB1 (universal bacterial marker) remained stable across both timepoints, indicating consistent sample quality and total microbial load (Fig. 7). A few participants, such as M8A and F9A/F10A, showed no detectable LR following treatment despite prior positivity, which could reflect issues related to non-compliance, or individual microbiota dynamics that prevent stable colonization.

Among participants who were *Lactobacillus reuteri* (LR)–negative at baseline, the estimated fold change in LR abundance following 10 days of probiotic treatment ranged from 70 × to 13,593 ×, with a mean ± SD of 3852 ± 4573. A paired two-tailed *t*-test comparing pre- and post-treatment CT values in these individuals showed a statistically significant reduction in CT values (*p* = 0.00012), indicating a robust increase in LR abundance post-intervention.

These values are now reported explicitly in the revised Results section, and the term “significant” has been appropriately supported with quantitative data and statistical analysis.

Overall, these results suggest that even short-term (10-day) probiotic administration can effectively and rapidly enhance gut colonization by *Lactobacillus reuteri*, particularly in individuals who were not previously colonized. The stronger colonization response observed in males may reflect sex-related microbiome or host interaction differences, warranting further investigation in larger cohorts.

## Discussion

Amyloid beta aggregation is central to the pathogenesis of AD, where the accumulation of fibrils and plaques contributes to neurotoxicity and cognitive decline [41, 42]. Inhibition of Aβ and Tau aggregation has therefore been a major focus for therapeutic interventions aimed at reducing or halting disease progression [43–46]. Our docking and AGGRESCAN analyses revealed that key probiotic proteins, such as ABC transporter permeases and d-Ala-d-Ala carboxypeptidases, interact with hotspot residues in Aβ_42_ (residues 17–42) and tau (PHF273–284 motif). These regions are crucial for β-sheet formation, oligomerization, and fibril elongation. Binding at these sites may sterically hinder or destabilize aggregation nuclei, thereby disrupting fibrillization pathways. These predicted interactions were validated with experimental ThT fluorescence and TEM data, which clearly demonstrate reduced fibril formation for both Aβ_42_ and PHF-tau peptides upon treatment with probiotic derivatives. The in vitro inhibition of aggregation strengthens the biological relevance of our docking results.

The present study demonstrates that a probiotic formulation, specifically composed of a mixture of *L. reuteri*, *L. paracasei*, and *L. rhamnosus*, and their derivatives, significantly inhibits the aggregation of Aβ_42_ peptide, a hallmark of AD. We observed that both live and lysed probiotic formulations, as well as their growth supernatant, significantly reduced Aβ_42_ and Tau aggregation, with lysed probiotics showing the highest inhibitory effect. These interactions may disrupt key amino acid hot spots responsible for Aβ_42_’s conversion from monomers to toxic oligomers and aggregates. Aβ_42_ aggregation is initiated by residues within the central hydrophobic core and C-terminal region of the peptide, with the sequence between residues 17–42 being particularly crucial for β-sheet formation and fibrillization [47]. Proteins secreted by these probiotics might interact with these critical regions, preventing the conformational changes required for aggregation. For instance, we show that *L. reuteri* 06 Protein 473 and *L. reuteri* 59 Protein 352 were predicted to bind to residues 32–42, which are important for β-sheet stacking and fibril formation [48]. By targeting these regions, probiotic-derived proteins are potentially capable of interrupting the aggregation process, which could slow or halt the progression of AD. Similarly, proteins from *L. paracasei* 05, such as Protein 151, are predicted to interact with residues 34–41, potentially blocking Aβ_42_’s structural transition into toxic aggregates.

Previous studies have indicated that gut microbiota can influence brain function and amyloid pathology through the gut-brain axis, a bidirectional communication system between the gastrointestinal system and the central nervous system (CNS) [49, 50]. Specific bacterial strains, including *Lactobacillus* and *Bifidobacterium*, have been shown to modulate immune responses, reduce neuroinflammation, and potentially alter amyloid aggregation [13, 51]. The findings of the present study indicate that Lactobacillus strains may exert inhibitory effects on the aggregation of Aβ_42_, suggesting the potential for probiotics to interfere with the formation of amyloid fibrils, which could provide a new avenue for the development of therapeutic strategies for AD.

It is noteworthy that lPB demonstrated the most pronounced inhibitory effect on fibril formation. This observation is consistent with the prior findings of Islam et al. [52], which demonstrated that bacterial components, such as lipoteichoic acid or peptidoglycans, may interact with amyloidogenic proteins, influencing their aggregation pathways [53]. Furthermore, in line with the findings of Amiri et al. [54], metabolites produced by probiotic bacteria, such as short-chain fatty acids, have been implicated in reducing amyloid deposition and promoting synaptic plasticity [55]. These results suggest that the disruption of probiotic cells releases bioactive molecules that can interact with Aβ_42_ and inhibit its pathological assembly into toxic aggregates.

The role of the gut microbiome in AD is a rapidly growing field of research, with emerging evidence that dysbiosis, or an imbalance in gut microbiota, may exacerbate amyloid pathology [56]. These findings align with our prior research underscoring the pivotal role of the gut microbiome in promoting health and longevity [57]. In a comprehensive review, we explored how environmental factors, including gut microbiota, shape aging trajectories and enhance resilience against chronic diseases, such as neurodegenerative disorders. Additionally, a systematic review by Quansah and colleagues [58] highlighted the therapeutic potential of specific Lactobacillus species in modulating gut-brain axis signaling and mitigating amyloid pathology, further supporting the results of the present study. Our findings contribute to the growing body of evidence, suggesting that specific probiotic interventions may not only help restore a healthy gut microbiota but also exert a protective effect against amyloid aggregation in the brain. This could be mediated via microbial metabolites or direct interaction of bacterial proteins with amyloidogenic peptides, as indicated by the findings of our study.

The significance of these interactions is underscored by the fact that Aβ_42_ oligomers, which form in the early stages of aggregation, are more neurotoxic than larger fibrillar structures. By preventing the formation of oligomers, probiotics may offer a novel therapeutic approach to AD, potentially mitigating neurotoxicity and cognitive decline associated with Aβ aggregation. The predicted interactions of probiotic proteins with both the N-terminal and C-terminal residues of Aβ_42_ suggest that these proteins can interfere with multiple steps of the aggregation process.

The inhibition of Aβ_42_ and PHF-tau aggregation by probiotic proteins adds to a growing body of evidence that probiotics can modulate neurodegenerative processes, not only through the gut-brain axis but also by directly interacting with pathological proteins like Aβ and tau [59]. Aggregation of Aβ_42_ and Tau is directly implicated in synaptic dysfunction, neuronal loss, and cognitive impairment in Alzheimer’s disease. By preventing or delaying aggregation at early nucleation sites, these probiotic proteins may reduce the formation of toxic oligomers and potentially mitigate neurotoxicity, offering a plausible mechanism for therapeutic benefit.

Studies [60] have indicated that the microbiome’s influence on neurodegenerative diseases may be partially attributed to the secretion of bioactive compounds capable of modulating protein misfolding and aggregation. This aligns with the broader therapeutic potential of probiotics in addressing proteinopathies by stabilizing misfolded proteins and preventing their aggregation.

Our findings, which demonstrate an increased abundance of *L. reuteri* in fecal samples following probiotic supplementation, are consistent with previous studies reporting the colonization potential and gut persistence of *L. reuteri* strains in humans [61]. The observed post-supplementation increase in *L. reuteri* levels aligns with reports that probiotic consumption can transiently modulate gut microbiota composition, particularly in older adults [62]. While the detection of *L. reuteri* DNA in fecal samples post-supplementation supports its transit through the gastrointestinal tract, we acknowledge that this does not confirm long-term colonization. A follow-up analysis over an extended period (e.g., 1 month) would be necessary to determine persistence and functional integration within the host microbiome. Together, these results not only validate the efficacy of our supplementation protocol but also align with a growing body of evidence suggesting that *L. reuteri* may play a role in modulating host neurocognitive and immunological functions via gut–brain axis interactions. The fecal qPCR analysis was included to provide foundational evidence that the probiotic strains under investigation can survive gastrointestinal passage, a prerequisite for exerting any modulatory effects on the gut–brain axis. While not directly addressing amyloid or tau aggregation, this component strengthens the translational relevance of the study by confirming strain viability in vivo.

Despite the promising findings, several limitations should be acknowledged. First, the sample size was relatively small (*n* = 20), which may limit the generalizability of the results and the ability to detect sex-specific differences in colonization patterns. Second, the study relied on short-term follow-up (10 days post-treatment), and longer-term persistence of *Lactobacillus reuteri* colonization remains to be determined. Third, although qPCR provided quantitative insights into bacterial abundance, it does not capture functional activity or strain-specific interactions within the broader gut microbiome. Finally, factors such as diet, host genetics, and baseline microbiota composition were not controlled or measured, which could influence probiotic efficacy and colonization outcomes.

The ability of probiotics and their derivatives to inhibit Aβ and Tau fibrillization in vitro provides a foundation for future research into their potential use in clinical settings. Further studies are needed to confirm whether these in vitro effects translate into meaningful cognitive benefits in vivo and to explore the molecular mechanisms underlying these interactions.

Top left: Surface representation of PA 5 protein 151 (light blue) docked with Aβ_42_ (magenta), showing a pronounced interaction groove. Top right: 3D model of PA 5 protein 151 (light blue) bound to full-length tau (green, backbone representation). The binding site localizes to a central cavity of the tau molecule. Bottom left: Atomic interactions between PA 5 151 (green) and Aβ_42_ (cyan), highlighting hydrogen bonds (yellow dashed lines) with key residues such as VAL-40, GLY-38, and LYS-394. Interaction distances are labelled in Å. Bottom right: Molecular interaction map between PA 5 151 (green) and tau (cyan), involving residues like PHE-301, THR-302, and GLU-303. The interactions suggest a possible inhibitory interface affecting tau aggregation or microtubule binding. Color key for all figures: light blue—bacterial protein 151; magenta—Aβ_42_ peptide; green (backbone)—Tau protein (full-length); green (sticks)—residues in binding site; cyan/blue (sticks)—human protein residues (Aβ_42_ or tau); yellow dashed lines—hydrogen/non-covalent interactions. Numbers (Å): Distance between interacting atoms.

Top left: Surface view of PA 6 protein 473 (light blue) bound to Aβ_42_ (magenta), illustrating a tight docking interface. Top right: Docking model of PA 6 protein 473 (light blue) with tau (green, backbone), positioned near a flexible loop region of tau. Bottom left: High-resolution interaction between PA 6 473 (green) and Aβ_42_ (cyan), involving TYR-288 and GLN-624. Strong hydrogen bonding and hydrophobic contacts are indicated. Bottom right: Zoomed-in view of PA 6 473 (green) interacting with tau (cyan), centered around residues ILE-32, VAL-21, and MET-25, forming a compact hydrogen bond network. Color key for all figures: light blue—bacterial protein 473; magenta—Aβ_42_ peptide; green (backbone)—Tau protein (full-length); green (sticks)—residues in binding site; cyan/blue (sticks)—human protein residues (Aβ_42_ or tau); yellow dashed lines—hydrogen/non-covalent interactions. Numbers (Å): Distance between interacting atoms.

Top left: PA 12 protein 1295 (light blue) complexed with Aβ_42_ (magenta) in a deep surface pocket. Top right: Full-length tau (green) bound to PA 12 1295 (light blue), demonstrating wide engagement across a beta-sheet–like domain. Bottom left: Close-up of the PA 12 1295 (green) and Aβ_42_ (cyan) interaction, showing bonding with residues GLU-22, GLY-25, and ILE-31. Multiple short-range contacts suggest high-affinity binding. Bottom right: Tau (cyan) and PA 12 1295 (green) interface highlighting residues THR-469, ILE-465, and ILE-307 in a tightly packed bonding environment. Color key for all figures: light blue—bacterial protein 1295; magenta—Aβ_42_ peptide; green (backbone)—Tau protein (full-length); green (sticks)—residues in binding site; cyan/blue (sticks)—human protein residues (Aβ_42_ or tau); Yellow dashed lines—hydrogen/non-covalent interactions. Numbers (Å): Distance between interacting atoms.

Top left: Surface model showing PA 59 protein 168 (light blue) with Aβ_42_ (magenta) bound along a central cleft. Top right: Docking model with tau (green) engaging the surface of PA 59 168 (light blue), indicating a widespread interaction zone. Bottom left: Detailed interactions between PA 59 168 (green) and Aβ_42_ (cyan) through residues ILE-363, VAL-40, PHE-15, and LEU-382. Interaction distances suggest effective stabilization. Bottom right: Molecular interaction between PA 59 168 (green) and tau (cyan), showing involvement of GLU-300, LYS-469, and PHE-301 with inter-residue distances labelled in angstroms. Color key for all figures: light blue—bacterial protein 168; magenta—Aβ_42_ peptide; green (backbone)—Tau protein (full-length); green (sticks)—residues in binding site; cyan/blue (sticks)—human protein residues (Aβ_42_ or tau); yellow dashed lines—hydrogen/non-covalent interactions. Numbers (Å): Distance between interacting atoms.

## Supplementary Information

Below is the link to the electronic supplementary material.

ESM 1 (20.6 KB DOCX)

## Data Availability

No datasets were generated or analysed during the current study.
