# Supplementary material for: Lactobacilli Probiotics Prevent Amyloid-Beta Fibril Formation In Vitro
Source: Probiotics Antimicrob Proteins. 2025 Oct 13;18(3):4640–59. doi: 10.1007/s12602-025-10776-z (PMC13176048; doi:10.1007/s12602-025-10776-z)
Supplement: Supplementary file 1 — (20.6 KB DOCX) [file 12602_2025_10776_MOESM1_ESM.docx]

**Genomic DNA Extraction from Whole Blood**

Genomic DNA was extracted from human whole blood using the *GeneJET Whole Blood Genomic DNA Purification Mini Kit* (Thermo Fisher Scientific, Cat. No. K0781), following the manufacturer’s instructions with minor modifications. Briefly, 200 μL of whole blood was mixed with 20 μL of Proteinase K solution and vortexed. Subsequently, 400 μL of Lysis Solution was added, and the mixture was vortexed thoroughly to ensure homogeneity. Samples were incubated at 56°C for 10 minutes to facilitate cell lysis.

Following lysis, 200 μL of 96–100% ethanol was added to the lysate, mixed by pipetting, and transferred to a GeneJET spin column. The column was centrifuged at 6,000 × g for 1 minute, and the flow-through was discarded. The column was washed sequentially with 500 μL of Wash Buffer I (with ethanol added) and 500 μL of Wash Buffer II (with ethanol added), with centrifugation at 8,000 × g for 1 minute and ≥20,000 × g for 3 minutes, respectively. To remove residual wash buffer, an additional centrifugation at ≥20,000 × g for 1 minute was performed.

DNA was eluted by applying 200 μL of Elution Buffer directly to the centre of the column membrane, followed by a 2-minute incubation at room temperature and centrifugation at 8,000 × g for 1 minute. Purified genomic DNA was either used immediately for downstream applications or stored at −20°C until further analysis. DNA concentration and purity were assessed using a NanoDrop spectrophotometer (Thermo Fisher Scientific).

**APOE Genotyping**

APOE genotyping was performed using purified genomic DNA extracted from whole blood, as described above. Genotyping focused on identifying the three common APOE alleles (ε2, ε3, and ε4) through TaqMan® SNP Genotyping Assays (Thermo Fisher Scientific, Scoresby, VIC, Australia). Each DNA sample was genotyped for two single nucleotide polymorphisms (SNPs): rs429358 (Assay ID: C__3084793_20) and rs7412 (Assay ID: C__904973_10), according to the manufacturer’s instructions.

Reactions were prepared using TaqMan Genotyping Master Mix and run on the QuantStudio™ 7 Pro Real-Time PCR System (Thermo Fisher Scientific, Scoresby, VIC, Australia). Allelic discrimination was automatically performed using QuantStudio Design and Analysis Software (v2.6.0), allowing the determination of APOE genotypes based on the combination of the two SNPs. This approach has been previously validated and applied in large-scale cohort studies investigating genetic risk factors in neurodegenerative diseases (e.g., Goozee et al., 2018).

# qPCR CT Analysis and Fold Change Estimates

| Sample_ID | Sex | CT_Before | CT_After | LR_Before_Positive | LR_After_Positive | CT_Delta | Baseline_Status | Fold_Change_Est |
| --- | --- | --- | --- | --- | --- | --- | --- | --- |
| M1 | Male | 30.11 | 22.34 | False | True | 7.77 | Negative at Baseline | 218.27 |
| M2 | Male | 34.27 | 20.66 | False | True | 13.610000000000003 | Negative at Baseline | 12503.12 |
| M3 | Male | 24.82 | 21.07 | True | True | 3.75 | Positive at Baseline | 13.45 |
| M4 | Male | 34.75 | 24.12 | False | True | 10.629999999999999 | Negative at Baseline | 1584.71 |
| M5 | Male | 34.23 | 24.88 | False | True | 9.349999999999998 | Negative at Baseline | 652.58 |
| M6 | Male | 22.59 | 24.14 | True | True | -1.5500000000000007 | Positive at Baseline | 0.34 |
| M7 | Male | 22.27 | 23.83 | True | True | -1.5599999999999987 | Positive at Baseline | 0.34 |
| M8 | Male | 20.32 |  | True | False |  | Positive at Baseline |  |
| M9 | Male | 27.87 | 22.49 | False | True | 5.380000000000003 | Negative at Baseline | 41.64 |
| M10 | Male | 34.12 | 21.32 | False | True | 12.799999999999997 | Negative at Baseline | 7131.55 |
| F1 | Female | 33.17 | 24.2 | False | True | 8.970000000000002 | Negative at Baseline | 501.46 |
| F2 | Female | 34.85 | 21.92 | False | True | 12.93 | Negative at Baseline | 7804.01 |
| F3 | Female | 35.79 | 22.81 | False | True | 12.98 | Negative at Baseline | 8079.22 |
| F4 | Female | 23.73 | 24.91 | True | True | -1.1799999999999997 | Positive at Baseline | 0.44 |
| F5 | Female | 23.87 | 23.27 | True | True | 0.6000000000000014 | Positive at Baseline | 1.52 |
| F6 | Female | 19.48 | 23.38 | True | True | -3.8999999999999986 | Positive at Baseline | 0.07 |
| F7 | Female | 34.9 | 36.14 | False | False | -1.240000000000002 | Negative at Baseline | 0.42 |
| F8 | Female | 21.81 | 24.35 | True | True | -2.5400000000000027 | Positive at Baseline | 0.17 |
| F9 | Female | 21.47 | 37.93 | True | False | -16.46 | Positive at Baseline | 0.0 |
| F10 | Female | 19.22 | 38.0 | True | False | -18.78 | Positive at Baseline | 0.0 |
